# Low self-control, perceived social support and internet gaming addiction: findings from an ethnicity minority region in China

**DOI:** 10.3389/fpsyt.2024.1458626

**Published:** 2024-09-05

**Authors:** Cui Meng, Li Quancai, Cui Kunjie, Xin Yanyu, Lama Wencai, Xia Yiwei

**Affiliations:** ^1^ Department of Sociology, School of Sociology and Psychology, Central University of Finance and Economics, Beijing, China; ^2^ Institute for Social Policy and Social Work, Research Institute of Social Development, Southwestern University of Finance and Economics, Chengdu, China; ^3^ Institute of Sociology, Research Institute of Social Development, Southwestern University of Finance and Economics, Chengdu, China; ^4^ Institute of Ethnic and Religious Studies, Sichuan Academy of Social Sciences, Chengdu, China; ^5^ School of Law, Southwestern University of Finance and Economics, Chengdu, China

**Keywords:** internet gaming addiction, low self-control, perceived social support, self-control theory, social support theory, Yi ethnic minority, adolescents

## Abstract

**Background:**

Adolescent Internet gaming addiction (IGA) is an pincreasing global concern. Drawing on self-control theory, social support theory, and problem behavior theory, this study aimed to examine the relationships between low self-control, perceived social support, and IGA among Chinese Yi and Non-Yi adolescents, with a focus on the moderating role of perceived social support and ethnic differences.

**Methods:**

A cross-sectional study was conducted among 1,997 adolescents (53.78% female, mean age 14.70 years) in Liangshan Yi Autonomous Prefecture, Sichuan Province, China, using a multi-stage cluster random sampling method. Participants completed questionnaires assessing IGA (Internet Gaming Disorder Scale-Short Form), low self-control (Low Self-Control Scale), and perceived social support (Multidimensional Scale of Perceived Social Support). Data were analyzed using descriptive statistics, t-tests, OLS regression, and the seemingly unrelated estimator (SUE).

**Results:**

Low self-control was positively associated with IGA (β = 0.35, p < 0.001), while perceived social support was negatively associated with IGA (β = -0.27, p < 0.001). Perceived social support, particularly from family (β = -0.43, p < 0.05) and significant others (β = -0.49, p < 0.01), moderated the relationship between low self-control and IGA. These associations were more salient among Yi adolescents compared to non-Yi adolescents.

**Conclusions:**

This study highlights the protective role of perceived social support, especially from family and significant others, in buffering the risk of low self-control on IGA. The findings extend self-control theory and social support theory, and provide empirical support for problem behavior theory in a cross-cultural context. The results underscore the importance of considering cultural contexts in understanding IGA and developing targeted interventions for ethnic minority adolescents.

## 1 Introduction

Adolescent Internet gaming addiction is an increasingly serious global problem. A recent large-scale meta-analysis provided a pooled global Internet gaming addiction prevalence of 7.02%, which has been increasing annually (1). In China, the problem seems to be particularly severe. According to a survey of 6,379 Chinese adolescent gamers, the detection rate of Internet gaming disorder was as high as 17% (2), consistent with the Expert Consensus on the Prevention and Treatment of Gaming Disorders in China (2019 Edition) (3). A recent study found that the prevalence of gaming disorder among Chinese ethnic minority adolescents was approximately 23.83%, indicating an alarmingly high incidence in this population (4). Addiction to the Internet among adolescents in China is expected to increase because of the rapid development of the Internet in China. According to recent statistics, the number of internet users in China was 1.092 billion by December 2023, accounting for about one fifth of the global internet users, and the group under the age of 20 accounted for about 18.5% (5). The Internet penetration reached as high as 96.8% among the Chinese youth and adolescent population (6). Research has shown that Internet addiction is associated with a range of adverse outcomes, including physical and mental health problems, decreased academic performance (7), and problem behaviors (8–10). Given the large scale of Chinese adolescent internet users, the high prevalence of Internet gaming addiction, especially among ethnic minority groups, and the profound negative impacts of Internet addiction, it is necessary to explore the influencing factors and underlying mechanisms of Internet addiction in this population.

Existing studies have identified multiple important factors associated with adolescent Internet addiction. Based on Gottfredson and Hirschi’s low self-control theory, empirical studies have found that lower self-control is associated with a higher likelihood of problematic or addictive internet behaviors among adolescents (11–13). Meanwhile, Cullen’s social support theory provides another perspective for understanding Internet addiction (14). Social support from various sources, such as family, friends, and teachers, has been found to significantly reduce the risk of Internet addiction among adolescents (15–17). However, few studies have explored the interaction between self-control and perceived perceived social support in the context of Internet gaming addiction. According to problem behavior theory, the interaction between individual characteristics and environmental factors may jointly shape adolescent problem behaviors (18). Furthermore, Cohen and Wills’ stress-buffering model of social support posits that social support may play a protective role primarily when individuals face stressors or risk factors (19). Applying these theories to the field of Internet gaming addiction, it suggests that self-control and perceived social support may not independently influence Internet gaming addiction; instead, perceived social support may moderate the relationship between self-control and Internet gaming addiction, buffering the negative impact of low self-control. However, this hypothesis requires direct examination.

Furthermore, existing studies on the relationship between perceived social support and Internet gaming addiction have primarily focused on general population samples or a single cultural context, with insufficient attention paid to ethnic minority groups (17, 20, 21). China has a diverse ethnic cultural landscape, and different groups may have variations in values, social norms, and other aspects, which could influence the functioning of social support (22, 23). In particular, some ethnic minority adolescents living in ethnic minority-concentrated areas may receive more culturally specific social support (24). Therefore, investigating the unique influencing mechanisms of Internet gaming addiction among ethnic minority adolescents is crucial for understanding the role of perceived social support and developing culturally sensitive prevention measures (25). However, relevant research remains limited.

To address these research gaps, the present study investigates the relationships between low self-control, perceived social support, and Internet gaming addiction among Yi and Non-Yi adolescents in Liangshan Yi Autonomous Prefecture, Sichuan Province, China, using a cross-sectional design. The Yi ethnic group, one of the largest ethnic minorities in China, has a distinct cultural background that may influence the patterns and mechanisms of adolescent Internet gaming addiction. The Yi people have traditionally lived in a hierarchical society organized around clans and villages, with a strong emphasis on collectivism and social harmony (22, 23). They have a rich history of oral literature, religious beliefs, and traditional arts, which have been passed down through generations (26). However, with rapid modernization and the increasing influence of mainstream Chinese culture, the Yi community is experiencing significant socioeconomic and cultural transitions (22). These unique cultural norms and changing social contexts may shape the way Yi adolescents perceive and utilize social support, as well as their susceptibility to Internet gaming addiction. Therefore, investigating the influencing mechanisms of Internet gaming addiction among Yi adolescents can provide valuable insights into the role of cultural factors in this issue.

We examine the main effects of low self-control and perceived social support on Internet gaming addiction, the moderating role of perceived social support, and potential differences between Yi and Non-Yi adolescents. The findings contribute to expanding the applicability of existing theories in different cultural contexts and provide empirical references for developing culturally sensitive prevention and intervention strategies for adolescent Internet gaming addiction.

## 2 Literature review

### 2.1 Low self-control and internet addiction: perspective from low self-control theory

Gottfredson and Hirschi proposed in their book “A General Theory of Crime” that lack of self-control is the core characteristic of criminal behavior (27). They argued that most criminal and deviant behaviors can be viewed as the pursuit of immediate and easy gratification or the seeking of short-term and fleeting pleasures by individuals. Consequently, those with relatively lower self-control are more likely to engage disproportionately in criminal and deviant behaviors. Notably, Gottfredson and Hirschi adopted a concept of crime based on behavioral characteristics rather than legal definitions. Many non-criminal behaviors, such as various accidents, drug abuse, bullying, and school misconduct, also fit their characterization of “bringing immediate benefits but with high subsequent costs” (27, 28). This theoretical perspective provides a unified view for understanding various problem behaviors. It emphasizes that whether it is crime in the legal sense or other types of violations or risky behaviors, they may all reflect deficits in individuals’ self-control. This deficit makes it difficult for individuals to resist temptation, leading them to be more easily driven by short-term benefits while ignoring the long-term consequences of their actions.

Numerous empirical studies in criminology support the explanatory power of the low self-control theory. Meta-analyses have found that low self-control is one of the strongest predictors of various antisocial behaviors, such as violence (29) and drug abuse (30). These findings indicate that low self-control is indeed a key psychological trait that drives individuals to engage in impulsive and deviant behaviors (30, 31).

In recent years, the low self-control theory has begun to be applied to explain adolescent Internet gaming addiction behaviors. Several studies across different countries and regions have found that low self-control is an important predictor of problematic Internet use among adolescents (11–14, 32). More specifically, the relationship between self-control and Internet addiction may be mediated by multiple psychological and behavioral factors. For instance, individuals with low self-control may be at higher risk of excessive Internet use due to more procrastination behaviors (11). Moreover, they may also be more likely to use the Internet to escape reality due to greater self-discrepancy and negative emotions, ultimately leading to more severe Internet addiction problems (12).

In summary, based on the low self-control theory and relevant empirical research, this study proposes the following hypothesis:

H1: Self-control is significantly negatively associated with adolescent Internet gaming addiction, i.e., the lower the level of self-control, the higher the degree of Internet gaming addiction.

### 2.2 Perceived social support and internet addiction: perspective from social support theory

In contrast to self-control, perceived social support is considered as an important protective factor against Internet addiction. Cullen’s Social Support Theory posits that social support refers to the information individuals obtain from social relationships, leading them to believe that they are cared for, loved, respected, and valued, and that they belong to a reciprocal social network (14). This theory emphasizes that social support can reduce life stress, enhance coping abilities, and thereby inhibit individuals’ criminal and deviant behaviors. A substantial body of empirical research has found that social support is a crucial protective factor against Internet addiction. For example, Gunuc and Dogan, using a sample of Turkish adolescents, found that higher levels of perceived social support were associated with lower tendencies of Internet addiction (33). Similarly, studies on Chinese adolescent samples have also reported a significant negative correlation between perceived social support and Internet addiction (16, 17).

Furthermore, the impact of social support on adolescent Internet gaming addiction may vary depending on its source. Social support can be categorized into family support, peer support, and significant other support (e.g., from teachers) based on the providers (34). These three types of support, encompassing the main social relationships in adolescents’ lives, play important roles in their psychological and behavioral development. However, the differential effects of social support from various sources on Internet gaming addiction have been understudied (20, 35). Therefore, we propose the following hypotheses:

H2: Perceived social support is significantly negatively associated with adolescent Internet gaming addiction, i.e., the more perceived social support received, the lower the degree of Internet gaming addiction.
H2a: Family support is significantly negatively associated with adolescent Internet gaming addiction.
H2b: Peer support is significantly negatively associated with adolescent Internet gaming addiction.
H2c: Support from significant others is significantly negatively associated with adolescent Internet gaming addiction.

### 2.3 The moderating role of perceived social support

Self-control theory and social support theory provide important perspectives for investigating adolescent Internet gaming addiction. Self-control theory emphasizes that lack of self-control is a key risk factor for Internet gaming addiction, while social support theory indicates that social support is a crucial protective resource against Internet gaming addiction. Cullen’s social support theory primarily focuses on the direct and mediating effects of social support on criminal and deviant behaviors (14). As Chouhy summarized, “Social support is deemed to reduce crime directly but also indirectly by affecting criminogenic factors such as social control, social learning, or strain and moderating their effect on crime” (36). However, social support theory pays relatively less attention to the moderating role of social support. Nevertheless, the stress-buffering model of social support proposed by Cohen and Wills provides a theoretical foundation for exploring the moderating role of social support (19). This model posits that social support exerts its protective function mainly when individuals face stress or risk factors, implying that social support may moderate the relationship between risk factors and negative outcomes. Applying this theory to the field of Internet gaming addiction research, we propose that perceived social support may moderate the relationship between self-control deficits (as a risk factor) and Internet gaming addiction. This hypothesis can also be supported by Jessor’s problem behavior theory (18), which suggests that adolescent problem behaviors stem from the interaction of personal factors (such as self-control) and environmental factors (such as social support). Accordingly, we speculate that low self-control and lack of social support may form an interactive effect, jointly influencing adolescent Internet gaming addiction.

Although few studies have directly examined the moderating effect of social support on the relationship between self-control and Internet gaming addiction, existing research has primarily focused on the direct and mediating effects of social support. For example, one study reported the partial mediating effect of social support on the relationship between self-efficacy, self-control, and Internet addiction, but did not consider its moderating role (15). However, some related studies have provided hints that social support may moderate the associations between other psychological or environmental factors and Internet addiction. For instance, a recent study on Hong Kong Chinese adolescents found that family support buffered the relationship between depression and Internet addiction (37). Of course, some studies challenge the view that social support invariably buffers people from the impacts of adverse factors. These studies indicate that adolescents with high levels of social support may use the Internet more for socializing, which can potentially lead to Internet addiction (38). This mixed research landscape also highlights the necessity of further exploring the potential moderating role of social support.

Theoretically, social support theory, as conceptualized in our measurement scale (the Multidimensional Scale of Perceived Social Support), encompasses various sources of social support, particularly family, peers, and significant others, which are important components of social support (34). These specific types of social support should also have moderating effects based on the stress-buffering model. This relationship has also received empirical support. Studies have shown that family support (39), peer support (40), and support from significant others (41) have moderating effects on children’s mental health and behavioral outcomes, although these studies may not directly focus on Internet gaming addiction. Considering the theoretical basis and related empirical evidence, we propose that different sources of social support, including family support, peer support, and support from significant others, may all have moderating effects on the relationship between self-control and Internet gaming addiction. However, the strength of these moderating effects may vary due to the different nature and functions of these social support sources, which requires further examination in the specific context of Internet gaming addiction.

Based on the aforementioned theories and empirical research, this study proposes the following hypotheses:

H3: Perceived social support moderates the relationship between self-control and Internet gaming addiction, i.e., the higher the level of perceived social support, the weaker the impact of self-control on Internet gaming addiction.
H3a: Family support moderates the relationship between self-control and Internet gaming addiction.
H3b: Peer support moderates the relationship between self-control and Internet gaming addiction.
H3c: Support from significant others moderates the relationship between self-control and Internet gaming addiction.

### 2.4 The heterogeneity effect of perceived social support among different ethnicity groups

The effect of perceived social support may vary due to cultural and ethnic differences (25). Individuals in collectivist cultures usually have closer ties with their families and communities, which may enhance the role of perceived social support (42). This cultural difference may be particularly prominent among adolescents from ethnic minority groups, especially for those living in ethnic minority-concentrated areas (22, 23). Compared to ethnic minority adolescents living in mainstream cultural areas, those living in areas with high ethnic minority concentration may have stronger ethnic identity and closer community ties, thus receiving more social support resources (24). For example, a study on Native American adolescents found that support from family members and tribal members could buffer the damage of racial discrimination on their self-esteem, while for Native American adolescents living in non-Native American communities, this protective effect was not evident (24). This suggests that ethnic minority enclaves may provide more culturally specific support resources, which are crucial for the mental health and adaptation of ethnic minority adolescents.

Based on the above analysis, we speculate that adolescents living in ethnic minority enclaves may have closer community ties and more culturally specific support resources. Therefore, perceived perceived social support may have a stronger protective effect on their Internet gaming addiction behaviors, manifested as a larger moderating effect of perceived social support on the relationship between self-control and Internet gaming addiction. To test this speculation, this study selected Yi adolescents in Liangshan Yi Autonomous Prefecture, Sichuan Province, China, as the research subjects. There are several considerations for choosing this group. First, the Yi ethnic group is one of the largest ethnic minorities in China, and Liangshan Prefecture is one of their main settlement areas. As of the end of 2023, Liangshan Prefecture had a Yi population of 3.0424 million, accounting for about 30% of China’s total Yi population (43). Therefore, the sample has good representativeness. Second, due to the harsh natural environment and previous history of tribal conflicts, the Yi communities in Liangshan have developed a strong collectivist culture based on cultural institutions such as family branches, in-laws, and villages, constructing a robust social support system (22, 23). This provides an ideal cultural context for examining the moderating effect of social support. Finally, Liangshan Prefecture is located in a remote mountainous area with relatively backward economic conditions. The rapid popularization of the Internet may bring unique adaptation challenges to local adolescents. Examining how social support helps them cope with the risk of Internet gaming addiction has important practical implications for formulating localized prevention measures.

In summary, this study aims to test the following hypotheses among Yi adolescents in Liangshan:

H4: The moderating effect of perceived social support on the relationship between self-control and Internet gaming addiction is more significant among ethnic minority adolescents.
H4a: The moderating effect of family support is stronger among ethnic minority adolescents.
H4b: The moderating effect of peer support is stronger among ethnic minority adolescents.
H4c: The moderating effect of support from significant others is stronger among ethnic minority adolescents.

## 3 Method

### 3.1 Data

Adopting a multi-stage cluster random sampling method, this study recruited 2,110 adolescents (grades 7–8) attending five secondary schools in Liangshan Yi Autonomous Prefecture, Sichuan Province, China. The multi-stage sampling process included the following stages.

In the first stage, five administrative regions within Liangshan Yi Autonomous Prefecture that met our study criteria were identified. Liangshan Yi Autonomous Prefecture is composed of 17 counties, with the Yi ethnic group divided into several cultural circles. Specifically, the Yi people in Liangshan are categorized into the “Shizhang” cultural circle, the “Suodi” cultural circle, the “Adu” cultural circle, and the “Rino” cultural circle. For our study, we selected one county from each of these cultural circles based on local Yi people’s perception of the county most representative of their cultural characteristics. Additionally, to enrich the diversity and comprehensiveness of our sample, we included one more county, which is known for its diverse indigenous ethnic groups and multi-ethnic cohabitation. These selections formed the five regions used in our study.

In the second stage, one secondary school within each selected region was chosen using the probability proportional to size (PPS) sampling method. The sampling design aimed to recruit 400 students from each selected school. Within each school, students in grades 7 and 8 were randomly selected to participate in the study. Access to the Internet and online games was a criterion for inclusion. The initial target was to have 400 students per school to ensure equal probability of selection across all individuals. However, due to practical constraints, the actual sample sizes from the five schools were 391, 418, 402, 410, and 376, respectively. To account for the discrepancies between the actual sample sizes and the targeted sample size, sampling weights were applied during the analysis.

Due to the presence of missing values, items with missing data were dropped with using listwise deletion, resulting in a final sample size of 1,997 (5.35% reduction). The final sample included 53.78% females, with an average age of 14.70 years (SD = 1.19). In terms of ethnicity, 82.52% of the participants were Yi ethnicity, and 17.48% are non-Yi ethnicity (5.96% were Han ethnicity and 11.52% were other ethnic minorities, including 4.45% Zang ethnicity, 2.41% Miao ethnicity, 1.94% Lisu ethnicity, 1.37% Menggu ethnicity, 0.52% Naxi ethnicity, 0.09% Hui ethnicity, 0.05% Zhuang ethnicity, and 0.47% who did not specify their ethnicity). The study was approved by research ethics committee from the PI’s university, and all respondents were informed of their rights of informed consent, voluntary participation, anonymity, and confidentiality.

### 3.2 Measure

Dependent Variable. Internet gaming addiction was measured by the Internet Gaming Disorder Scale-Short Form Pontes and Griffiths (44), which comprises 9 items assessing the degree of individuals’ addiction to Internet gaming. All 9 items were rated using a 5-point Likert-type scale (1=never and 5=very often), with a higher score on the scale indicating a higher risk of developing Internet gaming addiction. The 9-item scale has previously been validated with promising psychometric properties in English, Italian, Turkish, Persian, Portuguese, Albanian, and Chinese samples (45). The Cronbach’s alpha for the scale in our study was 0.87.

Independent Variable: Low self-control was measured using the Low Self-Control Scale developed by Grasmick and colleagues (46). This scale contains 24 items with 6 subscales (each with 4 items) measuring 6 dimensions of low self-control: impulsivity, simple tasks, risk-seeking, physical activity, self-centeredness, and temper. The items were rated using a 4-point Likert-type scale, ranging from strongly disagree to strongly agree. Higher scores on the scale indicate a higher likelihood of becoming angry and lower levels of self-control. The original version of the scale has demonstrated good explanatory power in a sample of Chinese adolescents (47), and the Cronbach’s alpha for the scale in our study was 0.90.

Moderating Variable: Perceived social support was measured using the Multidimensional Scale of Perceived Social Support (MSPSS) developed by Zimet and colleagues (34). This scale contains 12 items measuring three dimensions of perceived social support: family, friends, and significant other with each dimension comprising 4 items. The items were rated using a 7-point Likert-type scale, ranging from 1 (very strongly disagree) to 7 (very strongly agree). Higher scores on the scale indicate higher levels of perceived social support. The original version of the scale has demonstrated good psychometric properties in various samples. In our study, the Cronbach’s alpha for the overall scale was 0.93, with 0.79 for the family subscale, 0.86 for the friends subscale, and 0.86 for the significant other subscale.

Several control variables were included in this study, guided by problem behavior theory (18) and previous empirical findings. According to problem behavior theory, both individual and environmental factors can influence adolescents’ problem behaviors. Specifically, we controlled for gender (1 = female, 0 = male) and age (in years) as individual factors, as previous studies have shown that males and younger adolescents are more likely to develop Internet gaming addiction (48, 49). We also included ethnicity (1 = Yi ethnicity, 0 = other ethnicity) and hukou status (1 = rural, 0 = urban) to account for potential cultural and regional differences in Internet gaming behavior. Research has shown that rural hukou status is associated with higher rates of problematic Internet use among Chinese adolescents (50, 51). Quancai et al. (32) further emphasized the importance of considering cultural contexts in understanding Internet gaming addiction among adolescents. Regarding environmental factors, we controlled for family structure, including single-parent status (1 = living with a single parent, 0 = otherwise) and no-parent status (1 = not living with either parent, 0 = otherwise), as adolescents from non-intact families have been found to be at higher risk of Internet gaming addiction (52–54). Family socioeconomic status (SES), measured on a scale from 1 to 5 with higher scores indicating higher SES, was also included as an environmental factor, as lower family SES has been associated with higher levels of Internet gaming addiction among adolescents (51, 55).

### 3.3 Analytical method

Firstly, descriptive statistics were employed to summarize the characteristics of the studied variables. Subsequently, *t*-tests were conducted to examine the differences in key dependent, independent, and control variables between Yi ethnicity and non-Yi ethnicity groups. Then, OLS regression analyses were utilized to investigate the effects of low self-control and perceived social support on Internet gaming addiction, while controlling for other confounding variables. To explore the moderating effect of perceived social support on the relationship between low self-control and Internet gaming addiction, interaction terms were incorporated into the regression models. Additionally, given that perceived social support comprises three dimensions, this study assessed whether the main effects and moderating effects differed across these dimensions.

Moreover, to compare the effect of low self-control and perceived social support on Internet game addition between the Yi ethnicity and non-Yi ethnicity groups, separate regression analyses were conducted for each group. Recognizing the sample size disparity between the Yi ethnicity and non-Yi ethnicity groups, direct comparison of coefficients may not be reliable. Thus, the seemingly unrelated estimator (SUE) was employed to simultaneously estimate the regression outcomes for Yi and non-Yi ethnicity groups. This method allows for more accurate comparisons by accounting for the potential correlation between the error terms of the two groups, thereby mitigating the influence of sample size differences and providing a more robust comparison. Finally, to provide a more intuitive representation of the moderating effect, margins plots were utilized to depict the trends in the relationship between low self-control and Internet gaming addiction at varying levels of perceived social support (low, medium, high). All analyses were performed using STATA 16.0, with an alpha level of 0.05 set for statistical significance. Prior to conducting the regression analyses, the variance inflation factors (VIF) were calculated for all variables to assess the potential presence of multicollinearity. The VIF values for all variables in the main models were found to be less than 10, indicating that multicollinearity is not a significant concern (56, 57).

## 4 Results

**Table 1** provides a comprehensive overview of the descriptive statistics for the study’s variables. The dependent variable, Internet gaming addiction, shows a mean score of 12.88 (SD = 5.40), indicating a low level of Internet gaming addiction among the respondents. The independent variable, low self-control, has a mean score of 1.89 (SD = 0.48), which is relatively low within its possible range, suggesting the self-control of the respondents is relatively high. The moderating variables are assessed through various dimensions of perceived social support. Overall social support has a mean score of 4.98 (SD = 1.19), suggesting a moderate to high level of perceived social support. Similar statistics are also observed in family support (Mean = 5.16, SD = 1.30), support from friends (Mean = 4.76, SD = 1.31), and support from significant others (Mean = 5.02, SD = 1.29), all indicating moderate to high levels of perceived support.

**Table 1:** Descriptive analysis (N=1,997).

	Mean/%	Weighted Mean/%	Std. dev.	Min	Max
Dependent variable					
Internet addiction (IA)	12.88	12.88	5.40	9.00	45.00
Independent variable					
Low self-control (LSC)	1.89	1.89	0.48	1.00	4.00
Moderating variable					
Social support overall (SS-Overall)	4.98	4.98	1.19	1.00	7.00
Social support from family (SS-Fam)	5.16	5.17	1.30	1.00	7.00
Social support from friend (SS-Frd)	4.76	4.76	1.31	1.00	7.00
Social support from sig. others (SS-O)	5.02	5.02	1.29	1.00	7.00
Control variable					
Female	53.78%	53.94%			
Age	14.70	14.71	1.19	11.00	18.00
Grade=7	53.98%	53.91%			
Ethnicity= Yi	82.52%	82.89%			
Hukou=rural	95.54%	95.58%			
Single parent	13.12%	13.23%			
No parent	3.66%	3.64%			
Family SES	2.32	2.32	0.68	1	5.00

Weighted mean and percentage are calculated with sampling weights.

Control variables reveal that 53.94% of the sample are female, with a mean age of 14.71 years (SD = 1.19), ranging from 11 to 18 years. The distribution of grades indicates that 53.91% are in grade 7. Ethnic composition predominantly consists of Yi ethnicity at 82.89%, with 95.58% of respondents having rural hukou status. Additionally, 13.23% of respondents come from single-parent families, and 3.64% report having no parents. The family SES shows a mean score of 2.32 (SD = 0.68), with scores ranging from 1 to 5.

**Table 2** presents the t-test results of key variables by Yi and non-Yi ethnicity. The dependent variable, Internet gaming addiction, shows that non-Yi students exhibit higher levels of Internet gaming addiction, compared to Yi ethnicity students (t = 1.02, p < 0.01). For the independent variable, low self-control, non-Yi respondents demonstrate higher self-control compared to Yi respondents (t = -0.08, p < 0.01). Moderating variables, perceived social support, also show significant differences. Non-Yi respondents perceive higher overall social support (t = 0.43, p < 0.001), family support (t = 0.38, p < 0.001), support from friends (t = 0.50, p < 0.001), and support from significant others (t = 0.40, p < 0.001).

**Table 2:** *t*-test of key variables by Yi and non-Yi ethnicity.

	Yi ethnicity (N=1,648)	Non-Yi ethnicity (N=349)	Mean differences *t* Value
Dependent variable			
Internet addiction (IA)	12.71	13.73	1.02^**^
Independent variable			
Low self-control (LSC)	1.90	1.82	-0.08^**^
Moderating variable			
Social support overall (SS-Overall)	4.91	5.34	0.43^***^
Social support from family (SS-Fam)	5.11	5.49	0.38^***^
Social support from friend (SS-Frd)	4.67	5.17	0.50^***^
Social support from sig. others (SS-O)	4.95	5.36	0.40^***^
Control variable			
Female	0.56	0.45	-0.11^***^
Age	14.85	14.04	-0.81^***^
Grade=7	0.54	0.54	-0.00
Hukou=rural	0.96	0.95	-0.01
Single parent	0.13	0.13	0.00
No parent	0.04	0.01	-0.03^***^
Family SES	2.24	2.68	0.44^***^

^**^ p < 0.01, ^***^ p < 0.001. All results are calculated with sampling weights.

As for control variables, a higher proportion of Yi respondents are female compared to non-Yi respondents (t = -0.11, p < 0.001). Yi respondents are also older on average compared to non-Yi respondents (t = -0.81, p < 0.001). The no parent variable is slightly higher among Yi respondents (t = -0.03, p < 0.001). Family SES is significantly lower among Yi respondents compared to non-Yi respondents (t = 0.44, p < 0.001).

**Table 3** presents the zero-order correlation matrix among the key variables examined in our study. The table reveals that, apart from the high correlations among different types of social support, the correlations among other variables are not particularly high. This indicates that in subsequent regression analyses, different types of social support should be incorporated into the regression model separately and should not be included in the same model.

**Table 3:** Zero-order correlation.

		(1)	(2)	(3)	(4)	(5)	(6)	(7)	(8)	(9)	(10)	(11)	(12)	(13)	(14)
(1)	IA	1.00													
(2)	LSC	0.32^***^	1.00												
		(0.00)													
(3)	SS-Overall	-0.14^***^	-0.15^***^	1.00											
		(0.00)	(0.00)												
(4)	SS-Fam	-0.14^***^	-0.17^***^	0.90^***^	1.00										
		(0.00)	(0.00)	(0.00)											
(5)	SS-Frd	-0.09^***^	-0.08^***^	0.90^***^	0.69^***^	1.00									
		(0.00)	(0.00)	(0.00)	(0.00)										
(6)	SS-O	-0.15^***^	-0.16^***^	0.93^***^	0.76^***^	0.79^***^	1.00								
		(0.00)	(0.00)	(0.00)	(0.00)	(0.00)									
(7)	Female	-0.33^***^	-0.12^***^	0.01	0.02	-0.01	0.04	1.00							
		(0.00)	(0.00)	(0.51)	(0.50)	(0.77)	(0.12)								
(8)	Age	-0.05^*^	-0.02	-0.04	-0.06^*^	-0.04	-0.02	0.04	1.00						
		(0.03)	(0.27)	(0.05)	(0.01)	(0.10)	(0.27)	(0.08)							
(9)	Grade=7	-0.05^*^	-0.02	-0.00	0.03	-0.05^*^	0.00	0.03	-0.38^***^	1.00					
		(0.04)	(0.47)	(0.91)	(0.12)	(0.04)	(0.90)	(0.25)	(0.00)						
(10)	Ethnicity= Yi	-0.07^**^	0.06^**^	-0.13^***^	-0.11^***^	-0.14^***^	-0.12^***^	0.08^***^	0.25^***^	0.00	1.00				
		(0.00)	(0.00)	(0.00)	(0.00)	(0.00)	(0.00)	(0.00)	(0.00)	(0.96)					
(11)	Hukou=rural	0.02	-0.06^**^	0.04^*^	0.04	0.03	0.04	0.01	0.05^**^	0.05^*^	0.01	1.00			
		(0.37)	(0.01)	(0.05)	(0.06)	(0.11)	(0.05)	(0.51)	(0.00)	(0.04)	(0.68)				
(12)	Single parent	-0.04	-0.00	-0.03	-0.02	-0.05^*^	-0.01	0.04	0.04	-0.03	0.00	-0.04	1.00		
		(0.10)	(0.98)	(0.20)	(0.37)	(0.02)	(0.80)	(0.05)	(0.10)	(0.15)	(0.95)	(0.14)			
(13)	No parent	-0.01	0.01	-0.02	-0.01	-0.03	-0.02	0.03	0.03	-0.03	0.07^***^	-0.04	0.29^***^	1.00	
		(0.46)	(0.53)	(0.30)	(0.56)	(0.26)	(0.32)	(0.16)	(0.20)	(0.17)	(0.00)	(0.22)	(0.00)		
(14)	Family SES	0.04^*^	-0.01	0.10^***^	0.08^**^	0.12^***^	0.09^***^	-0.12^***^	-0.12^***^	0.03	-0.24^***^	-0.02	-0.14^***^	-0.14^***^	1.00
		(0.05)	(0.83)	(0.00)	(0.00)	(0.00)	(0.00)	(0.00)	(0.00)	(0.20)	(0.00)	(0.34)	(0.00)	(0.00)	

^*^ p < 0.05, ^**^ p < 0.01, ^***^ p < 0.001. All correlation coefficients are estimated with sampling weights.

The table also shows a significant positive correlation between low self-control and Internet gaming addiction (r = 0.32, p < 0.001). The perceived overall social support (r = -0.14, p < 0.001), social support from family (r = -0.14, p < 0.001), social support from friends (r = -0.09, p < 0.01), and social support from significant others (r = -0.15, p < 0.001) show significant negative correlations with Internet gaming addiction.

**Table 4** presents the regression results for the overall sample, focusing on the effects of low self-control, perceived social support, and their interactions on Internet gaming addiction (See **Supplementary Table S1** in the appendixes for all coefficients). Firstly, across all models, low self-control consistently shows a significant positive effect on Internet gaming addiction (all p < 0.001). This effect remains significant in all subsequent models, demonstrating that lower self-control is robustly associated with higher levels of Internet gaming addiction, supporting H1.

**Table 4:** Regression for overall sample.

	(1)	(2)	(3)	(4)	(5)	(6)	(7)	(8)	(9)
LSC	3.24^***^	3.08^***^	5.76^***^	3.08^***^	5.67^***^	3.17^***^	4.74^***^	3.07^***^	5.71^***^
	(0.28)	(0.28)	(1.20)	(0.28)	(1.03)	(0.28)	(1.12)	(0.28)	(1.19)
SS-Overall		-0.46^***^	0.49						
		(0.10)	(0.40)						
LSC×SS-Overall			-0.51^*^						
			(0.23)						
SS-Fam				-0.37^***^	0.52				
				(0.10)	(0.32)				
LSC×SS-Fam					-0.48^*^				
					(0.19)				
SS-Frd						-0.35^***^	0.22		
						(0.09)	(0.39)		
LSC×SS-Frd							-0.31		
							(0.23)		
SS-O								-0.43^***^	0.51
								(0.10)	(0.39)
LSC×SS-O									-0.50^*^
									(0.22)
Control variables		Yes	Yes	Yes	Yes	Yes	Yes	Yes	Yes
Constant	11.66^***^	14.20^***^	9.05^**^	13.89^***^	8.92^***^	13.45^***^	10.49^***^	13.93^***^	8.85^**^
	(1.73)	(1.87)	(2.80)	(1.86)	(2.52)	(1.85)	(2.64)	(1.83)	(2.78)
*R* ^2^	0.203	0.214	0.217	0.212	0.216	0.210	0.212	0.213	0.217
adj. *R* ^2^	0.199	0.209	0.212	0.207	0.211	0.206	0.207	0.209	0.212
*AIC*	11970.24	11946.68	11940.38	11951.33	11943.22	11954.75	11953.35	11947.17	11940.31
*BIC*	12031.84	12013.87	12013.17	12018.52	12016.01	12021.94	12026.15	12014.36	12013.10
F	36.72	38.27	35.88	36.77	35.61	36.47	34.21	38.24	35.59
*N*	1997	1997	1997	1997	1997	1997	1997	1997	1997

LSC stands for low self-control, SS-Overall stands for social support overall, SS-Fam stands for social support from family, SS-Frd stands for social support from friend, SS-O stands for social support from sig. others. Standard errors in parentheses, *p < 0.05, **p < 0.01, ***p < 0.001. All models controlled for sex=female, age, Grade=7, Ethnicity= Yi, Hukou=rural, single parent, no parents, and family SES. All regression coefficients are estimated with sampling weights.

Additionally, perceived social support in various forms, including overall social support, family support, support from friends, and support from significant others, consistently shows a negative effect on Internet gaming addiction (all p < 0.001). This indicates that higher levels of perceived social support are generally associated with lower levels of Internet gaming addiction, supporting H2 and H2a-H2c.

Secondly, the moderating effects of perceived social support on the relationship between low self-control and Internet gaming addiction may not apply uniformly across all types of perceived social support. The interaction terms reveal that overall social support (b = -0.51, p < 0.05), family support (b = -0.48, p < 0.05), and support from significant others (b = -0.50, p < 0.05) significantly moderate the relationship between low self-control and Internet gaming addiction. This suggests that these forms of perceived social support can buffer the negative impact of low self-control on Internet gaming addiction, supporting H3, H3a, and H3c. However, the interaction term for support from friends is not significant, indicating that support from friends does not have a significant moderating effect on this relationship, thus not supporting H3b.

**Table 5** presents the regression results for different ethnic groups using both Naïve and SUE (See **Supplementary Tables S2**, **S3** in the appendixes for all coefficients and VIFs in the Naïve models). Firstly, the main effects of low self-control and various forms of perceived social support are significant for the Yi ethnicity group (all p < 0.001). However, for the non-Yi ethnicity group, only the main effect of low self-control is significant; the main effects of various forms of perceived social support are not significant. This result remains consistent even when using the SUE.

**Table 5:** Regression for different ethnicity group by Naïve and Seemingly uncorrelated estimator.

	Naïve estimator				Seemingly uncorrelated estimator			
	Yi ethnicity		Non-Yi ethnicity		Yi ethnicity		Non-Yi ethnicity	
LSC	2.94^***^	6.19^***^	4.20^***^	5.29	2.93^***^	6.18^***^	4.20^***^	5.29
	(0.24)	(0.99)	(0.70)	(3.52)	(0.29)	(1.25)	(0.88)	(4.08)
SS-Overall	-0.48^***^	0.69	-0.39	-0.02	-0.48^***^	0.69	-0.39	-0.02
	(0.10)	(0.36)	(0.27)	(1.20)	(0.11)	(0.42)	(0.28)	(1.33)
LSC×SS-Overall		-0.63^***^		-0.19		-0.63^**^		-0.19
		(0.19)		(0.60)		(0.24)		(0.75)
LSC	2.95^***^	5.97^***^	4.11^***^	4.90	2.95^***^	5.97^***^	4.11^***^	4.90
	(0.24)	(0.86)	(0.71)	(3.30)	(0.29)	(1.04)	(0.89)	(3.65)
SS-Fam	-0.36^***^	0.68^*^	-0.45	-0.19	-0.36^***^	0.68^*^	-0.45	-0.19
	(0.08)	(0.30)	(0.25)	(1.11)	(0.10)	(0.32)	(0.28)	(1.17)
LSC×SS-Fam		-0.57^***^		-0.13		-0.57^**^		-0.13
		(0.16)		(0.55)		(0.19)		(0.64)
LSC	3.03^***^	5.16^***^	4.31^***^	3.94	3.03^***^	5.16^***^	4.31^***^	3.94
	(0.24)	(0.91)	(0.70)	(3.10)	(0.29)	(1.19)	(0.87)	(3.60)
SS-Frd	-0.37^***^	0.43	-0.25	-0.37	-0.37^***^	0.43	-0.25	-0.37
	(0.09)	(0.34)	(0.24)	(1.04)	(0.10)	(0.42)	(0.24)	(1.18)
LSC×SS-Frd		-0.43^*^		0.07		-0.43		0.07
		(0.18)		(0.54)		(0.25)		(0.68)
LSC	2.91^***^	5.82^***^	4.25^***^	7.02^*^	2.91^***^	5.82^***^	4.25^***^	7.02
	(0.24)	(0.94)	(0.70)	(3.34)	(0.29)	(1.25)	(0.87)	(3.70)
SS-O	-0.46^***^	0.59	-0.31	0.62	-0.46^***^	0.59	-0.31	0.62
	(0.09)	(0.34)	(0.25)	(1.13)	(0.10)	(0.41)	(0.26)	(1.22)
LSC×SS-O		-0.56^**^		-0.49		-0.56^*^		-0.49
		(0.17)		(0.57)		(0.24)		(0.68)

LSC stands for low self-control, SS-Overall stands for social support overall, SS-Fam stands for social support from family, SS-Frd stands for social support from friend, SS-O stands for social support from sig. others. Robust standard errors in parentheses, *p < 0.05, **p < 0.01, ***p < 0.001, All models controlled for sex=female, age, Grade=7, Hukou=rural, single parent, no parents, and family SES. All regression coefficients are estimated with sampling weights.

Secondly, the moderating effects of various forms of perceived social support are significant only for the Yi ethnicity group (all p < 0.05). However, the interaction with support from friends is not significant under the SUE, suggesting a less consistent moderating effect for this type of perceived social support. In contrast, for non-Yi respondents, none of the perceived social support interactions are significant, indicating that perceived social support does not moderate the relationship between low self-control and Internet gaming addiction in this group. This pattern remains consistent even when using the SUE.

In summary, H4 is partially supported. Perceived social support moderates the relationship between low self-control and Internet gaming addiction more significantly among minority youth groups, particularly in terms of overall social support, social support from family, and social support from significant others, supporting H4, H4a, and H4c. However, the moderating effect of support from friends does not show a significant difference, indicating that H4b is not supported.

**Figure 1:**
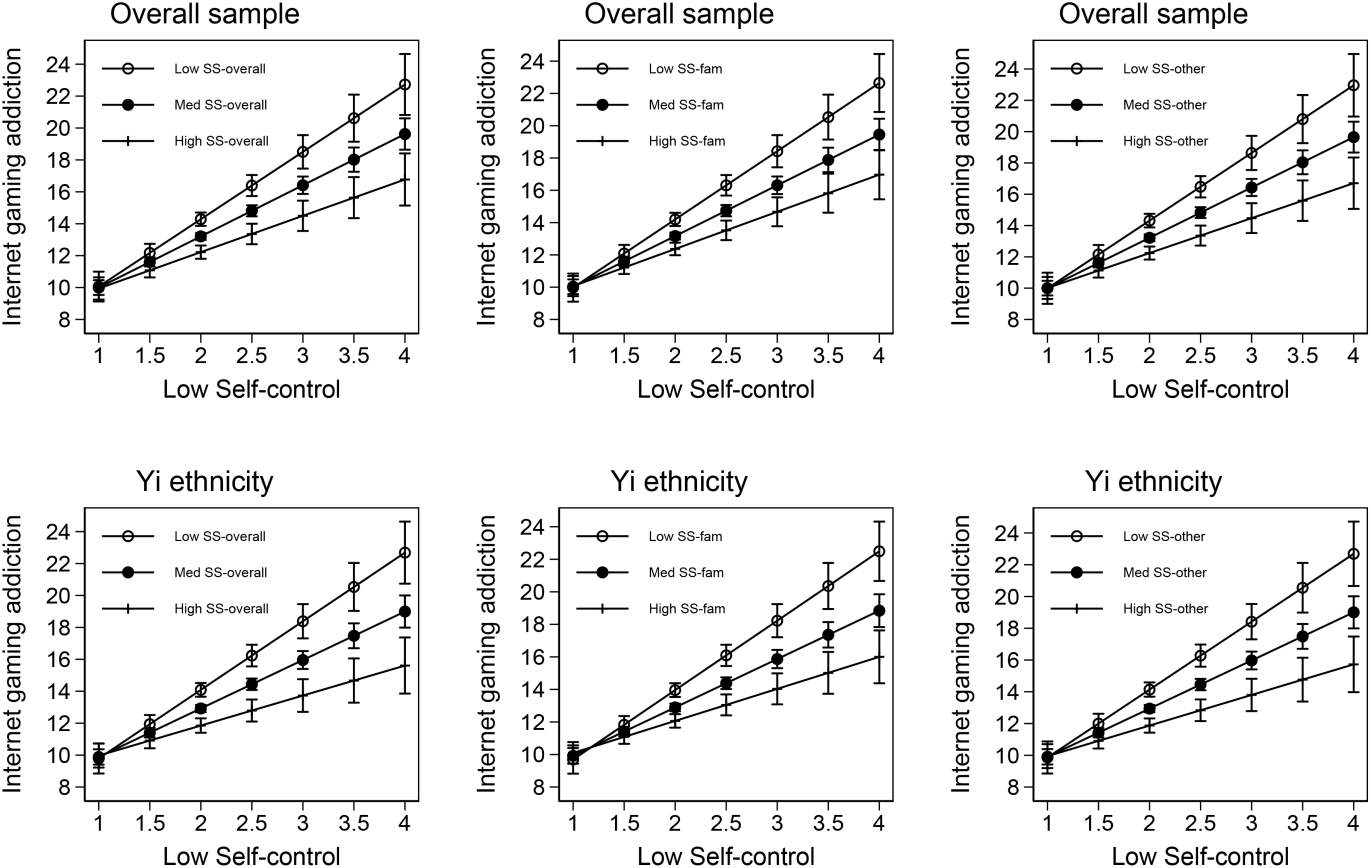
Interaction plot.

**Figure 1** illustrates the moderating effects of perceived social support on the relationship between low self-control and Internet gaming addiction using margins plot. The figure consists of six panels, with the first row representing the overall sample and the second row representing the Yi ethnicity group. The first column shows the moderating effect of overall social support, the second column depicts the moderating effect of social support from family, and the third column displays the moderating effect of social support from significant others.

From the figure, it is evident that when low self-control is very low (indicating high self-control), the level of Internet gaming addiction remains relatively low regardless of the level of perceived social support. However, as the level of perceived social support decreases, the slope of the relationship between low self-control and Internet gaming addiction becomes steeper, indicating a more pronounced increase in Internet gaming addiction. Conversely, when perceived social support is high, the slope is attenuated, suggesting a weaker association between low self-control and Internet gaming addiction. This pattern holds true for both the overall sample and the Yi ethnicity group, and it is consistent across different types of perceived social support, including overall social support, social support from family, and social support from significant others.

Finally, among the control variables, gender (β = -3.12, p < 0.001), ethnicity (β = -2.33, p < 0.001), and hukou status (β = 1.09, p < 0.01) were significantly associated with Internet gaming addiction. Specifically, females, Yi adolescents, and urban adolescents reported lower levels of Internet gaming addiction.

## 5 Discussion

This study investigated the relationships between low self-control, perceived social support, and Internet gaming addiction among middle school students from Yi and Han ethnic groups in Southwest China. It also examined the moderating role of perceived social support and its source differences, and considered the manifestation of these relationships in different cultural backgrounds (ethnic minority and non-ethnic minority). The results showed that low self-control was positively correlated with Internet gaming addiction, while perceived social support was negatively correlated with Internet gaming addiction. Perceived social support, especially family support and support from significant others, could significantly buffer the impact of low self-control on Internet gaming addiction. However, the moderating role of friend support was not significant. These relationships were more pronounced in the ethnic minority sample. The study, for the first time, systematically compared the differentiated roles of social support from different sources in moderating the relationship between low self-control and Internet gaming addiction in a cross-cultural context, providing a new empirical basis for an integrated theoretical model of individual traits, environmental factors, and cultural backgrounds.

This study first verified the influence of low self-control and perceived social support on adolescent Internet gaming addiction. Regarding low self-control, we found that it is an important influencing factor of Internet gaming addiction, which is consistent with the core viewpoint of Gottfredson and Hirschi’s general theory of crime, that is, lack of self-control is the root of a series of deviant and criminal behaviors including addiction (27). Multiple studies have verified the association between low self-control and adolescent Internet addiction or problematic Internet use in different cultural contexts (12, 13). This study, using a sample of adolescents from ethnic minority areas in Southwest China, further supports the universality of this theoretical perspective. In terms of perceived perceived social support, this study found that it was significantly negatively correlated with Internet gaming addiction, providing new evidence for Cullen’s social support theory in a cross-cultural context (14). This finding is consistent with the protective role of social support found in other regions of China (16, 17), indicating that regardless of whether they are mainstream or ethnic minority adolescents, obtaining good social support helps reduce their risk of Internet gaming addiction.

Furthermore, this study revealed that the relationships between different dimensions of perceived social support and Internet gaming addiction differ, which has been less discussed in previous studies that treated perceived social support as a single construct. Specifically, compared to friend support, family support and support from significant others had more significant negative correlations with Internet gaming addiction, and their moderating effects on the impact of low self-control were also more robust. This suggests that the role of perceived social support may vary depending on its source and nature.

The significant role of family support for adolescent development can be understood from multiple theoretical perspectives. Attachment theory emphasizes that stable and lasting parent-child emotional bonds are the foundation of an individual’s psychological development (58). Parental support implies a deeper sense of security and self-worth, which can enhance adolescents’ ability to resist addictive behaviors (59). Social control theory points out that close ties with traditional social agents such as family can inhibit individuals’ deviant behaviors (60). Existing research has found that parental support can significantly reduce adolescents’ level of Internet addiction (53). For example, a study targeting Turkish adolescents found that parental support, rather than peer support, could significantly predict Internet addiction (33). Although friends play an increasingly important role in adolescents’ social lives, family support may still be a more critical factor influencing adolescents’ Internet behavior. Additionally, the role of peers in Internet addiction is uncertain, as peers can be either a protective factor or a risk factor, which may be related to the Internet addiction rate in the peer environment (61).

In addition to family support, support from significant others also demonstrated a significant role in predicting Internet gaming addiction, even surpassing friend support. This finding may be related to the specific cultural background of this study. The sample of this study comes from a remote mountainous area in Southwest China. The local Yi culture has traditionally had a strong hierarchical system, with tribal leaders and elders holding important authority in the community (23). Moreover, as a typical agricultural civilization, the local culture also emphasizes social harmony and respect for authority (43). This cultural background may make the normative role of parental and significant other (such as teacher) support more prominent. The research suggests that when constructing theoretical models of adolescent Internet gaming addiction, it should be examined in the broader context of interpersonal relationships and cultural backgrounds. This is of great value for refining the mechanism of social support and formulating localized prevention and intervention strategies.

In addition to the main effects, this study also found that perceived social support can moderate the relationship between low self-control and Internet gaming addiction, which has important theoretical significance. Specifically, the results of this study provide an important supplement to Cullen’s social support theory (14). Social support theory mainly focuses on the direct impact of social support on deviant behaviors, while less exploring its moderating effect. This study shows that social support can also play an indirect protective role by buffering risk factors such as low self-control. This expands the explanatory power of social support theory, suggesting that we should examine social support in interaction with other factors to better understand its mechanism. At the same time, this finding also corroborates Cohen and Wills’ stress-buffering model of social support (19). This model emphasizes that social support mainly plays a protective role when individuals face risk factors, and this study found that perceived social support can buffer the impact of low self-control on Internet gaming addiction, providing new empirical support for this model. Furthermore, this finding also echoes and deepens Jessor’s problem behavior theory (18). This theory emphasizes that adolescent problem behaviors are the result of the interaction of personal factors (such as self-control) and environmental factors (such as social support). This study not only supports the basic assumption of this theory but also empirically clarifies the internal mechanism of the interaction of personal and environmental factors influencing problem behaviors. That is, social support can buffer the negative impact of low self-control, thereby reducing the risk of Internet gaming addiction. This finding enriches and expands the connotation of problem behavior theory.

Lastly, this study also explored the differences in the role of perceived social support across different groups. We found that the moderating effect of perceived social support on the relationship between self-control and Internet gaming addiction was more significant among Yi adolescents. This result may reflect the importance of cultural and socioeconomic backgrounds in shaping the function of social support. Compared to mainstream cultural adolescents, Yi adolescents live in a cultural environment that places more emphasis on collectivist values and stresses close ties between individuals and their families and communities, making them more likely to receive support from significant others (22). More importantly, the sample of this study shows that the family SES of Yi adolescents is significantly lower than that of non-Yi adolescents. Lower family SES may imply greater developmental stress and adaptation challenges. According to Cohen and Wills’ stress-buffering model, social support has a stronger protective effect on disadvantaged groups in high-stress and low-resource states (19). Yi adolescents, who face greater developmental stress and adaptation challenges, may need and benefit more from social support. This study thereby provides useful references for developing culturally and contextually sensitive theories of adolescent mental health (25).

Although this study initially hypothesized that Yi adolescents living in ethnic minority enclaves might have closer community ties and more culturally specific support resources, the data analysis revealed that perceived social support levels among Yi adolescents were significantly lower than those of non-Yi adolescents. This discrepancy may stem from the following reasons: First, despite the cohesive culture within Yi communities, the Yi group as a whole is in a relatively impoverished state compared to non-Yi groups, as reflected in their lower family socioeconomic status (see **Table 2**). The constraints of socioeconomic conditions may affect the quality of social support available to Yi adolescents. Second, in the context of Liangshan Yi Autonomous Prefecture, non-Yi people constitute a minority, accounting for only 17.48% of the total sample (see **Table 1**). Social identity theory (62) suggests that minority group members may strengthen their ingroup identity and cohesion when facing survival pressures or marginalization. This could lead to enhanced social support, self-esteem, and sense of security within the group (63–65). Consequently, non-Yi adolescents, as a minority in this Yi-dominated area, may form stronger mutual support networks to cope with adaptation challenges, explaining their higher reported levels of social support. This finding underscores the importance of considering factors such as socioeconomic conditions and minority group survival strategies when examining social support among adolescents in ethnic minority enclaves.

The findings of this study have important implications for the prevention and intervention of adolescent Internet gaming addiction. First, the results emphasize the importance of enhancing adolescents’ self-control ability. Schools and families can enhance adolescents’ self-control ability by designing targeted training programs, such as time management and emotion regulation strategy courses. Second, the study highlights the key role of social support, especially family support and support from significant others. This suggests that preventive measures should focus more on the family system, such as conducting family education courses to improve parents’ support ability and Internet literacy. Especially for Yi and other ethnic minority adolescents, intervention measures should fully consider their cultural characteristics, such as utilizing the traditional family and community support networks to design culturally appropriate prevention programs. Considering the buffering effect of social support on low self-control, for adolescents with weaker self-control ability, more efforts should be made to build a strong social support system to reduce their risk of Internet gaming addiction.

Although this study provides new perspectives for understanding adolescent Internet gaming addiction, it still has some limitations. The primary one is that this study adopted a cross-sectional design, which limits our inference of the causal relationships between variables. Future research can adopt a longitudinal design to track the developmental trajectory of adolescents’ Internet use behaviors, more accurately revealing the dynamic relationships between self-control, perceived social support, and Internet gaming addiction. Second, the sample of this study mainly comes from the Liangshan Yi Autonomous Prefecture in Sichuan Province, which may limit the generalizability of the results. Subsequent research can expand the sample range to include adolescents from more regions and ethnicities to test the cross-cultural stability of the results.

## 6 Conclusion

This study, by examining the relationships between low self-control, perceived social support, and adolescent Internet gaming addiction, as well as the differences in these relationships among Yi and Non-Yi adolescent groups, provides a new perspective for understanding the issue of adolescent Internet gaming addiction. The research results not only verify the main effects of low self-control and perceived social support on Internet gaming addiction but, more importantly, reveal the moderating role of perceived social support in buffering the impact of low self-control on Internet gaming addiction. This finding provides an empirical basis for integrating self-control theory, social support theory, and problem behavior theory, emphasizing the importance of the interaction between individual traits and environmental factors.

Particularly noteworthy is that this study found cultural differences in the role of social support, which was more prominent in the Yi adolescent group. This result highlights the necessity of considering cultural backgrounds when studying adolescent problem behaviors, providing important insights for developing culturally sensitive theories and intervention strategies. Moreover, the study identified the differentiated effects of social support from different sources, providing a new direction for refining the understanding of social support mechanisms.

These findings have direct guiding significance for the prevention and intervention practices of adolescent Internet gaming addiction. They emphasize the importance of simultaneously focusing on enhancing self-control ability and strengthening social support systems in intervention strategies, especially when designing programs targeting ethnic minority adolescents, where their unique cultural backgrounds and social support networks should be fully considered.

## Data Availability

The raw data supporting the conclusions of this article will be made available by the authors, without undue reservation.

## Supplementary material

Supplementary Table 1 Regression for overall sample Note: LSC stands for low self-control, SS-Overall stands for social support overall, SS-Fam stands for social support from family, SS-Frd stands for social support from friend, SS-O stands for social support from sig. others. Standard errors in parentheses, * p < 0.05, ** p < 0.01, *** p < 0.001. All models controlled for sex=female, age, Grade=7, Ethnicity= Yi, Hukou=rural, single parent, no parents, and family SES. All regression coefficients are estimated with sampling weights.

Supplementary Table 2 Regression for Yi ethnicity Note: LSC stands for low self-control, SS-Overall stands for social support overall, SS-Fam stands for social support from family, SS-Frd stands for social support from friend, SS-O stands for social support from sig. others. Standard errors in parentheses, * p < 0.05, ** p < 0.01, *** p < 0.001. All models controlled for sex=female, age, Grade=7, Ethnicity= Yi, Hukou=rural, single parent, no parents, and family SES. All regression coefficients are estimated with sampling weights.

Supplementary Table 3 Regression for non-Yi ethnicity Note: LSC stands for low self-control, SS-Overall stands for social support overall, SS-Fam stands for social support from family, SS-Frd stands for social support from friend, SS-O stands for social support from sig. others. Standard errors in parentheses, * p < 0.05, ** p < 0.01, *** p < 0.001. All models controlled for sex=female, age, Grade=7, Ethnicity= Yi, Hukou=rural, single parent, no parents, and family SES. All regression coefficients are estimated with sampling weights.
