# Supplementary material for: Low self-control, perceived social support and internet gaming addiction: findings from an ethnicity minority region in China
Source: Front Psychiatry. 2024 Sep 5;15:1458626. doi: 10.3389/fpsyt.2024.1458626 (PMC11413865; doi:10.3389/fpsyt.2024.1458626)
Supplement: Supplementary Table 1 — Regression for overall sample Note: LSC stands for low self-control, SS-Overall stands for social support overall, SS-Fam stands for social support from family, SS-Frd stands for social support from friend, SS-O stands for social support from sig. others. Standard errors in parentheses, * p < 0.05, ** p < 0.01, *** p < 0.001. All models controlled for sex=female, age, Grade=7, Ethnicity= Yi, Hukou=rural, single parent, no parents, and family SES. All regression coefficients are estimated with sampling weights. [file Table1.docx]

**Appendix**

Table S1 Regression for overall sample

|  | (1) | (2) | (3) | (4) | (5) | (6) | (7) | (8) | (9) |
| --- | --- | --- | --- | --- | --- | --- | --- | --- | --- |
| LSC | 3.24^***^ | 3.08^***^ | 5.76^***^ | 3.08^***^ | 5.67^***^ | 3.17^***^ | 4.74^***^ | 3.07^***^ | 5.71^***^ |
|  | (0.28) | (0.28) | (1.20) | (0.28) | (1.03) | (0.28) | (1.12) | (0.28) | (1.19) |
| SS-Overall |  | -0.46^***^ | 0.49 |  |  |  |  |  |  |
|  |  | (0.10) | (0.40) |  |  |  |  |  |  |
| LSC×SS-Overall |  |  | -0.51^*^ |  |  |  |  |  |  |
|  |  |  | (0.23) |  |  |  |  |  |  |
| SS-Fam |  |  |  | -0.37^***^ | 0.52 |  |  |  |  |
|  |  |  |  | (0.10) | (0.32) |  |  |  |  |
| LSC×SS-Fam |  |  |  |  | -0.48^*^ |  |  |  |  |
|  |  |  |  |  | (0.19) |  |  |  |  |
| SS-Frd |  |  |  |  |  | -0.35^***^ | 0.22 |  |  |
|  |  |  |  |  |  | (0.09) | (0.39) |  |  |
| LSC×SS-Frd |  |  |  |  |  |  | -0.31 |  |  |
|  |  |  |  |  |  |  | (0.23) |  |  |
| SS-O |  |  |  |  |  |  |  | -0.43^***^ | 0.51 |
|  |  |  |  |  |  |  |  | (0.10) | (0.39) |
| LSC×SS-O |  |  |  |  |  |  |  |  | -0.50^*^ |
|  |  |  |  |  |  |  |  |  | (0.22) |
| Female | -3.14^***^ | -3.12^***^ | -3.12^***^ | -3.12^***^ | -3.13^***^ | -3.13^***^ | -3.13^***^ | -3.10^***^ | -3.09^***^ |
|  | (0.22) | (0.22) | (0.22) | (0.22) | (0.22) | (0.22) | (0.22) | (0.22) | (0.22) |
| Age | -0.11 | -0.12 | -0.11 | -0.12 | -0.11 | -0.12 | -0.11 | -0.11 | -0.10 |
|  | (0.09) | (0.09) | (0.09) | (0.09) | (0.09) | (0.09) | (0.09) | (0.09) | (0.09) |
| Grade=7 | -0.45 | -0.46^*^ | -0.43 | -0.42 | -0.39 | -0.49^*^ | -0.48^*^ | -0.44 | -0.43 |
|  | (0.23) | (0.23) | (0.23) | (0.23) | (0.23) | (0.23) | (0.23) | (0.23) | (0.23) |
| Ethnicity= Yi | -2.25^***^ | -2.33^***^ | -2.32^***^ | -2.29^***^ | -2.28^***^ | -2.33^***^ | -2.33^***^ | -2.33^***^ | -2.33^***^ |
|  | (0.62) | (0.62) | (0.62) | (0.62) | (0.62) | (0.62) | (0.63) | (0.62) | (0.62) |
| Hukou=rural | 0.99^*^ | 1.09^**^ | 1.14^**^ | 1.08^**^ | 1.11^**^ | 1.06^**^ | 1.09^**^ | 1.08^**^ | 1.13^**^ |
|  | (0.42) | (0.41) | (0.41) | (0.41) | (0.41) | (0.41) | (0.41) | (0.41) | (0.41) |
| Single parent | -0.32 | -0.35 | -0.34 | -0.33 | -0.34 | -0.37 | -0.37 | -0.31 | -0.30 |
|  | (0.32) | (0.32) | (0.32) | (0.32) | (0.32) | (0.32) | (0.32) | (0.32) | (0.32) |
| No parent | 0.11 | 0.12 | 0.12 | 0.13 | 0.13 | 0.14 | 0.16 | 0.10 | 0.06 |
|  | (0.53) | (0.52) | (0.52) | (0.52) | (0.51) | (0.53) | (0.53) | (0.52) | (0.52) |
| Family SES | -0.08 | -0.01 | -0.04 | -0.03 | -0.05 | -0.02 | -0.04 | -0.02 | -0.06 |
|  | (0.18) | (0.18) | (0.18) | (0.18) | (0.18) | (0.18) | (0.18) | (0.18) | (0.18) |
| Constant | 11.66^***^ | 14.20^***^ | 9.05^**^ | 13.89^***^ | 8.92^***^ | 13.45^***^ | 10.49^***^ | 13.93^***^ | 8.85^**^ |
|  | (1.73) | (1.87) | (2.80) | (1.86) | (2.52) | (1.85) | (2.64) | (1.83) | (2.78) |
| *R*^2^ | 0.203 | 0.214  0.209  11946.68  12013.87  38.27  1997 | 0.217  0.212  11940.38  12013.17  35.88  1997 | 0.212  0.207  11951.33  12018.52  36.77  1997 | 0.216 | 0.210  0.206  11954.75  12021.94  36.47  1997 | 0.212  0.207  11953.35  12026.15  34.21  1997 | 0.213  0.209  11947.17  12014.36  38.24  1997 | 0.217  0.212  11940.31  12013.10  35.59  1997 |
| adj. *R*^2^ | 0.199  11970.24  12031.84  36.72  1997 |  |  |  | 0.211  11943.22  12016.01  35.61  1997 |  |  |  |  |
| *AIC* |  |  |  |  |  |  |  |  |  |
| *BIC* |  |  |  |  |  |  |  |  |  |
| F |  |  |  |  |  |  |  |  |  |
| *N* |  |  |  |  |  |  |  |  |  |

Note: LSC stands for low self-control, SS-Overall stands for social support overall, SS-Fam stands for social support from family, SS-Frd stands for social support from friend, SS-O stands for social support from sig. others. Standard errors in parentheses, ^*^ p < 0.05, ^**^ p < 0.01, ^***^ p < 0.001. All models controlled for sex=female, age, Grade=7, Ethnicity= Yi, Hukou=rural, single parent, no parents, and family SES. All regression coefficients are estimated with sampling weights. The range of VlF values shows no multicollinearity issue.

Table S2 Regression for Yi ethnicity

|  | (1) | (2) | (3) | (4) | (5) | (6) | (7) | (8) |
| --- | --- | --- | --- | --- | --- | --- | --- | --- |
| LSC | 2.94^***^ | 6.19^***^ | 2.95^***^ | 5.97^***^ | 3.03^***^ | 5.16^***^ | 2.91^***^ | 5.82^***^ |
|  | (0.24) | (0.99) | (0.24) | (0.86) | (0.24) | (0.91) | (0.24) | (0.94) |
| SS-Overall | -0.48^***^ | 0.69 |  |  |  |  |  |  |
|  | (0.10) | (0.36) |  |  |  |  |  |  |
| LSC×SS-Overall |  | -0.63^***^ |  |  |  |  |  |  |
|  |  | (0.19) |  |  |  |  |  |  |
| SS-Fam |  |  | -0.36^***^ | 0.68^*^ |  |  |  |  |
|  |  |  | (0.08) | (0.30) |  |  |  |  |
| LSC×SS-Fam |  |  |  | -0.57^***^ |  |  |  |  |
|  |  |  |  | (0.16) |  |  |  |  |
| SS-Frd |  |  |  |  | -0.37^***^ | 0.43 |  |  |
|  |  |  |  |  | (0.09) | (0.34) |  |  |
| LSC×SS-Frd |  |  |  |  |  | -0.43^*^ |  |  |
|  |  |  |  |  |  | (0.18) |  |  |
| SS-O |  |  |  |  |  |  | -0.46^***^ | 0.59 |
|  |  |  |  |  |  |  | (0.09) | (0.34) |
| LSC×SS-O |  |  |  |  |  |  |  | -0.56^**^ |
|  |  |  |  |  |  |  |  | (0.17) |
| Female | -2.93^***^ | -2.92^***^ | -2.95^***^ | -2.95^***^ | -2.95^***^ | -2.95^***^ | -2.91^***^ | -2.89^***^ |
|  | (0.23) | (0.23) | (0.23) | (0.23) | (0.23) | (0.23) | (0.23) | (0.23) |
| Age | -0.08 | -0.07 | -0.08 | -0.07 | -0.08 | -0.08 | -0.06 | -0.06 |
|  | (0.10) | (0.10) | (0.10) | (0.10) | (0.10) | (0.10) | (0.10) | (0.10) |
| Grade=7 | -0.30 | -0.28 | -0.26 | -0.22 | -0.35 | -0.33 | -0.29 | -0.27 |
|  | (0.25) | (0.24) | (0.25) | (0.25) | (0.25) | (0.25) | (0.25) | (0.24) |
| Hukou=rural | 1.22^*^ | 1.27^*^ | 1.22^*^ | 1.25^*^ | 1.19^*^ | 1.24^*^ | 1.23^*^ | 1.28^*^ |
|  | (0.56) | (0.56) | (0.56) | (0.56) | (0.56) | (0.56) | (0.56) | (0.56) |
| Single parent | -0.44 | -0.41 | -0.43 | -0.42 | -0.48 | -0.45 | -0.39 | -0.37 |
|  | (0.35) | (0.35) | (0.35) | (0.35) | (0.36) | (0.35) | (0.35) | (0.35) |
| No parent | 0.27 | 0.25 | 0.28 | 0.27 | 0.30 | 0.31 | 0.24 | 0.19 |
|  | (0.60) | (0.59) | (0.60) | (0.59) | (0.60) | (0.60) | (0.60) | (0.59) |
| Family SES | 0.18 | 0.13 | 0.16 | 0.13 | 0.18 | 0.14 | 0.17 | 0.12 |
|  | (0.17) | (0.17) | (0.17) | (0.17) | (0.17) | (0.17) | (0.17) | (0.17) |
| Constant | 10.91^***^ | 4.82 | 10.45^***^ | 4.80 | 10.25^***^ | 6.32^*^ | 10.65^***^ | 5.17^*^ |
|  | (1.90) | (2.61) | (1.90) | (2.45) | (1.89) | (2.49) | (1.88) | (2.54) |
| *R*^2^ | 0.206 | 0.211 | 0.202 | 0.209 | 0.202 | 0.205 | 0.206  0.202  9774.34  9828.47  47.56  1657 | 0.211  0.207  9766.05  9825.59  44.07  1657 |
| adj. *R*^2^ | 0.202 | 0.207 | 0.198 | 0.204 | 0.198 | 0.201 |  |  |
| *AIC* | 9775.61 | 9766.04 | 9782.43 | 9771.12 | 9782.88 | 9778.97  9838.51 |  |  |
| *BIC* | 9829.73 | 9825.58 | 9836.55 | 9830.66 | 9837.01 |  |  |  |
| F | 47.38 | 44.07 | 46.44 | 43.43 | 46.37 | 42.45  1657 |  |  |
| *N* | 1657 | 1657 | 1657 | 1657 | 1657 |  |  |  |

Note: LSC stands for low self-control, SS-Overall stands for social support overall, SS-Fam stands for social support from family, SS-Frd stands for social support from friend, SS-O stands for social support from sig. others. Standard errors in parentheses, ^*^ p < 0.05, ^**^ p < 0.01, ^***^ p < 0.001. All models controlled for sex=female, age, Grade=7, Ethnicity= Yi, Hukou=rural, single parent, no parents, and family SES. All regression coefficients are estimated with sampling weights. The range of VlF values shows no multicollinearity issue.

Table S3 Regression for non-Yi ethnicity

|  | (1) | (2) | (3) | (4) | (5) | (6) | (7) | (8) |
| --- | --- | --- | --- | --- | --- | --- | --- | --- |
| LSC | 4.20^***^ | 5.29 | 4.11^***^ | 4.90 | 4.31^***^ | 3.94 | 4.25^***^ | 7.02^*^ |
|  | (0.70) | (3.52) | (0.71) | (3.30) | (0.70) | (3.10) | (0.70) | (3.34) |
| SS-Overall | -0.39 | -0.02 |  |  |  |  |  |  |
|  | (0.27) | (1.20) |  |  |  |  |  |  |
| LSC×SS-Overall |  | -0.19 |  |  |  |  |  |  |
|  |  | (0.60) |  |  |  |  |  |  |
| SS-Fam |  |  | -0.45 | -0.19 |  |  |  |  |
|  |  |  | (0.25) | (1.11) |  |  |  |  |
| LSC×SS-Fam |  |  |  | -0.13 |  |  |  |  |
|  |  |  |  | (0.55) |  |  |  |  |
| SS-Frd |  |  |  |  | -0.25 | -0.37 |  |  |
|  |  |  |  |  | (0.24) | (1.04) |  |  |
| LSC×SS-Frd |  |  |  |  |  | 0.07 |  |  |
|  |  |  |  |  |  | (0.54) |  |  |
| SS-O |  |  |  |  |  |  | -0.31 | 0.62 |
|  |  |  |  |  |  |  | (0.25) | (1.13) |
| LSC×SS-O |  |  |  |  |  |  |  | -0.49 |
|  |  |  |  |  |  |  |  | (0.57) |
| Female | -3.90^***^ | -3.91^***^ | -3.89^***^ | -3.91^***^ | -3.89^***^ | -3.88^***^ | -3.88^***^ | -3.90^***^ |
|  | (0.63) | (0.64) | (0.63) | (0.63) | (0.64) | (0.64) | (0.63) | (0.63) |
| Age | -0.81^*^ | -0.80^*^ | -0.84^*^ | -0.83^*^ | -0.78^*^ | -0.79^*^ | -0.83^*^ | -0.79^*^ |
|  | (0.35) | (0.36) | (0.35) | (0.35) | (0.35) | (0.36) | (0.35) | (0.36) |
| Grade=7 | -1.52^*^ | -1.50^*^ | -1.54^*^ | -1.52^*^ | -1.52^*^ | -1.53^*^ | -1.54^*^ | -1.50^*^ |
|  | (0.71) | (0.72) | (0.71) | (0.72) | (0.72) | (0.72) | (0.71) | (0.72) |
| Hukou=rural | 0.54 | 0.56 | 0.60 | 0.61 | 0.45 | 0.44 | 0.46 | 0.52 |
|  | (1.45) | (1.45) | (1.45) | (1.45) | (1.45) | (1.46) | (1.45) | (1.45) |
| Single parent | -0.07 | -0.10 | -0.07 | -0.10 | -0.04 | -0.02 | -0.05 | -0.13 |
|  | (0.94) | (0.95) | (0.94) | (0.95) | (0.94) | (0.95) | (0.94) | (0.95) |
| No parent | -1.84 | -1.75 | -1.68 | -1.65 | -1.86 | -1.90 | -1.88 | -1.65 |
|  | (3.42) | (3.44) | (3.42) | (3.43) | (3.43) | (3.45) | (3.43) | (3.44) |
| Family SES | -1.28^*^ | -1.28^*^ | -1.27^*^ | -1.27^*^ | -1.33^*^ | -1.34^*^ | -1.30^*^ | -1.29^*^ |
|  | (0.58) | (0.58) | (0.57) | (0.58) | (0.58) | (0.58) | (0.58) | (0.58) |
| Constant | 25.11^***^ | 22.76^*^ | 25.88^***^ | 24.20^**^ | 23.82^***^ | 24.62^**^ | 24.90^***^ | 18.91^*^ |
|  | (6.09) | (9.63) | (6.11) | (9.21) | (5.98) | (8.84) | (6.13) | (9.35) |
| *R*^2^ | 0.236 | 0.236  0.213  2169.59  2211.77  10.23  342 | 0.238  0.218  2166.53  2204.87  11.55  342 | 0.239  0.216  2168.46  2210.65  10.37  342 | 0.233  0.213  2168.81  2207.16  11.23  342 | 0.233  0.210  2170.80  2212.98  10.08  342 | 0.234  0.214  2168.30  2206.65  11.30  342 | 0.236  0.213  2169.56  2211.74  10.23  342 |
| adj. *R*^2^ | 0.215  2167.69  2206.04  11.38  342 |  |  |  |  |  |  |  |
| *AIC* |  |  |  |  |  |  |  |  |
| *BIC* |  |  |  |  |  |  |  |  |
| F |  |  |  |  |  |  |  |  |
| *N* |  |  |  |  |  |  |  |  |

Note: LSC stands for low self-control, SS-Overall stands for social support overall, SS-Fam stands for social support from family, SS-Frd stands for social support from friend, SS-O stands for social support from sig. others. Standard errors in parentheses, ^*^ p < 0.05, ^**^ p < 0.01, ^***^ p < 0.001. All models controlled for sex=female, age, Grade=7, Ethnicity= Yi, Hukou=rural, single parent, no parents, and family SES. All regression coefficients are estimated with sampling weights. The range of VlF values shows no multicollinearity issue.
